# Single-cell RNA-sequencing of dermal fibroblasts demonstrates culture-induced changes and variable persistence of keloid disease features

**DOI:** 10.1016/j.isci.2026.115630

**Published:** 2026-04-07

**Authors:** Amy Lock, Elena M. Drudi, Dasha Freydina, Brian M. Stramer, Franziska Denk, Tanya J. Shaw

**Affiliations:** 1 Centre for Inflammation Biology and Cancer Immunology, School of Immunology and Microbial Sciences, King’s College London, London, UK; 2 Wolfson Sensory, Pain and Regeneration Centre (SPaRC), School of Neuroscience, King’s College London, London, UK; 3 Single Cell Omics Platform, Centre for Inflammation Biology and Cancer Immunology, School of Immunology and Microbial Sciences, King’s College London, London, UK; 4 Randall Centre for Cell and Molecular Biophysics, King’s College London, London, UK

**Keywords:** Molecular biology, Cell biology

## Abstract

*In vitro* models of scarring and fibrosis are essential to improve our understanding of disease mechanisms and ultimately develop much-needed therapeutic strategies. The emerging appreciation of fibroblast heterogeneity leaves a knowledge gap about what is represented in typical fibroblast cultures: are the quantitative differences in fibroblast subtypes observed in pathological tissues represented, and are disease-associated molecular alterations of subtypes maintained? Single-cell (sc) RNA-seq on patient-matched keloid and adjacent non-lesional dermis was compared to sc- and bulk-RNA-seq of fibroblast cultures after 4+ passages. After culture, fibroblast subtypes assimilated, with clustering distinct from the original populations. Pseudo-bulk analysis of non-cultured mesenchymal fibroblasts showed cell-intrinsic keloid-versus-control transcriptional differences consistent with disease understanding; however, only a subset of these persisted *in vitro*. Cell-cell communication analysis provides insight into potential strategies to maintain cell populations and their *in vivo* phenotypes. This work provides a greater understanding of, and potential strategies to refine, essential human fibroblast culture models.

## Highlights

• Fibroblast culture leads to subtype assimilation; heterogeneity persists minimally
• Culture induces dramatic phenotypic drift, but is not global to all signature genes
• Cell-cell communication analysis may hold clues for maintaining cell populations
• Comparing keloid to control pre/post-culture shows strengths and limitations of the model

## Graphical abstract

**Figure:**
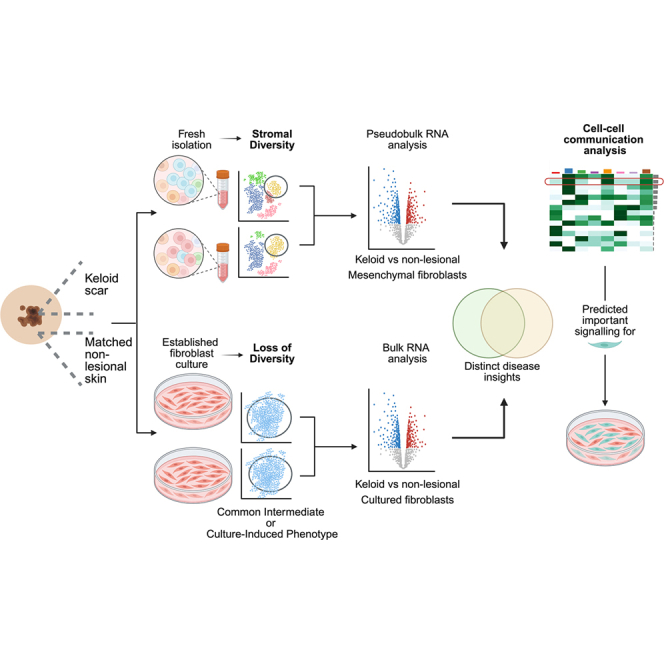

## Introduction

Keloids are a type of pathological scar characterized by abnormal collagen deposition and expansion beyond the wound boundary. They continue to grow over time, recur after resection, and do not regress spontaneously.^1^ As such, these scars can result in sizable masses with significant aesthetic concerns.^2^ Keloids are highly fibrotic and share many characteristics of fibrosis found in other areas of the body; such characteristics include a highly aligned architecture,^3^ fibroblast accumulation, and excessive and high collagen I:III ratio,^4^ leading to stiff tissue that is no longer able to function as adequately as its normal counterparts. Together with the ease of access to these surface-based lesions, keloids are an excellent model condition to study the biology of fibrosis.

Recently, several single-cell RNA sequencing (scRNA-seq) studies have compared cell type variety and abundance in keloids compared to normal scar and skin.^5^^,^^6^^,^^7^ These studies, individually and when considered collectively,^8^ have revealed distinct cell subsets to be enriched in keloids, such as extracellular matrix (ECM)-producing fibroblasts, among others. Building on these findings, developing *in vitro* models to study these cell types and their mechanisms is essential for advancing keloid research. As there is no suitable animal model that faithfully recapitulates keloid disease, the ability to expand primary cells is paramount; but there is concern about loss of *in vivo* phenotype^9^^,^^10^^,^^11^ and phenotypic drift.^12^ The recent scRNA-seq publications illustrating diverse cell composition in keloids indicate that it would be beneficial to be able to isolate and maintain particular (sub)populations for detailed mechanistic work.

In this study, we investigated the transcriptional changes that primary dermal cells isolated from keloid or adjacent non-lesional skin undergo during standard fibroblast culture conditions by scRNA-seq. We show that passage (P)4 cells are transcriptionally different from their *in vivo* (P0) derivatives, with changes in cell subset identity and proportions, and show variable effects on disease-associated transcriptional differences between keloid and non-lesional fibroblasts. We also provide insight into how culture conditions can be altered based on cell-cell communication analysis to shift cultured fibroblasts toward a particular (mesenchymal-like) phenotype.

## Results

### Dermal cell subsets are transcriptionally different by the 4th passage

**Figure 1:**
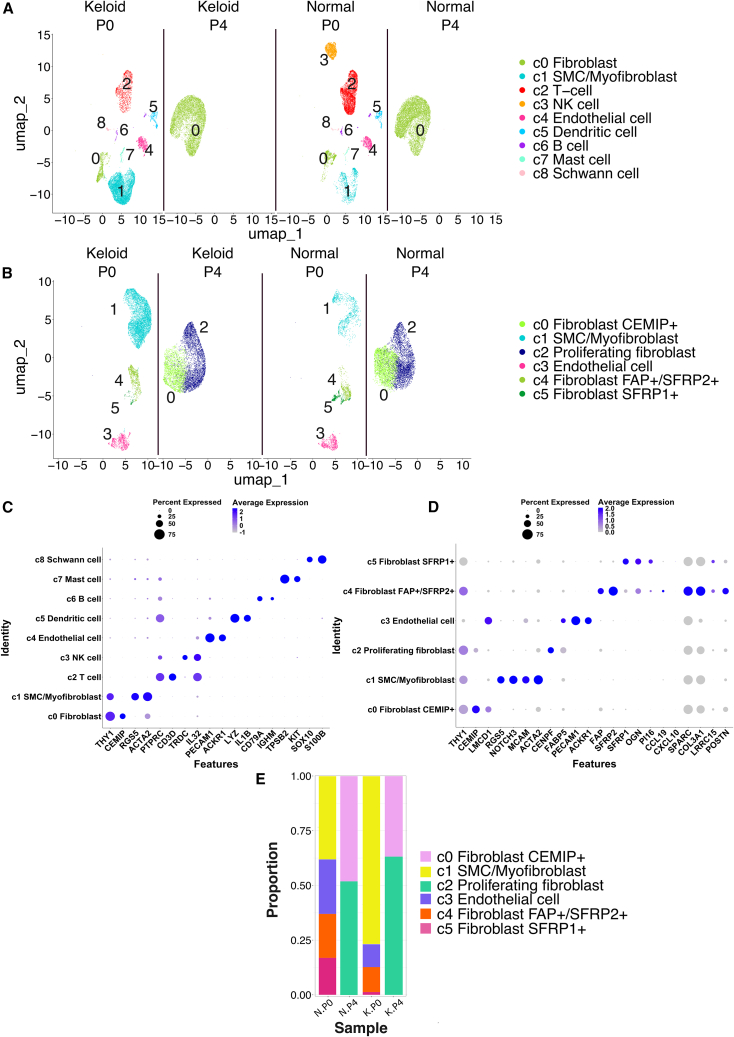
Single-cell RNA sequencing reveals that dermal cell subsets are transcriptionally different by the 4^th^ passage in culture (A and B) Sample-separated UMAPs of the unsupervised clustering of (A) all cells (*N* = 34,948) revealed 9 clusters and (B) stromal cells only (*N* = 25,956) revealed 6 clusters. P0 = passage 0, P4 = passage 4. (C and D) Expression of cluster marker genes across each cluster. Blue color gradient represents the average expression of the given gene. Circle size represents the percentage of cells within a cluster expressing a given gene. (E) Proportion of each stromal subset out of total stromal cells for normal primary dermal cells at passage 0 (N.P0) and passage 4 (N.P4), and keloid dermal cells at passage 0 (K.P0) and passage 4 (K.P4).

To improve our understanding of the extent of changes to dermal cell subsets over passage, we performed scRNA-seq to compare acutely isolated cells (P0) with those cultured over four passages (P4). Dermal cells were extracted from one male donor’s abdominal keloid scar and patient-matched adjacent non-lesional skin. Consistent with published studies,^5^^,^^6^^,^^7^ unsupervised clustering revealed nine cell types (Figures 1A and 1C). In this particular sample, smooth muscle cells (SMCs)/myofibroblasts represented the majority of keloid cells, with fibroblasts and endothelial cells representing similar proportions to each other (∼10%). Surprisingly, the most numerous cell types in normal adjacent skin were T-cells and NK cells (>50%; far exceeding that expected of normal skin), potentially reflecting an active immune response at the lesion margin in this patient.

There were stark differences in the cell types present at P4 compared to P0 for both keloid and non-lesional skin, with only cells identified as fibroblasts still present (Figure 1A). Selective unsupervised re-clustering of the stromal cells across time-points revealed six sub-clusters, which were annotated as: CEMIP^+^ fibroblast (c0), SMC/myofibroblast (c1), proliferating fibroblast (c2), endothelial cell (c3), FAP^+^/SFRP2^+^ fibroblast (c4), and SFRP1^+^ fibroblast (c5) (Figures 1B and D). Both non-lesional and keloid P4 cells were remarkably transcriptionally different from the four cell subsets present at P0, with only two distinct clusters observed: CEMIP^+^ fibroblasts and proliferating fibroblasts (Figure 1E). These cell populations were additionally annotated according to “atlas datasets” describing universal fibroblasts in health and disease.^13^^,^^14^ Using the markers of fibroblasts in healthy tissues, the FAP+/SFRP2+ subset was delineated by SPARC/COL3A1, whereas no subsets were marked by CCL19/CXCL10, indicating a lack of a proinflammatory subset in this particular sample (Figure 1D). Interestingly, the FAP+/SFRP2+ subset expressed key markers also observed in universal perturbed fibroblasts (LRRC15, POSTN) (Figure 1D).

### Passaged cells represent transcriptional assimilation of *in vivo* subsets

**Figure 2:**
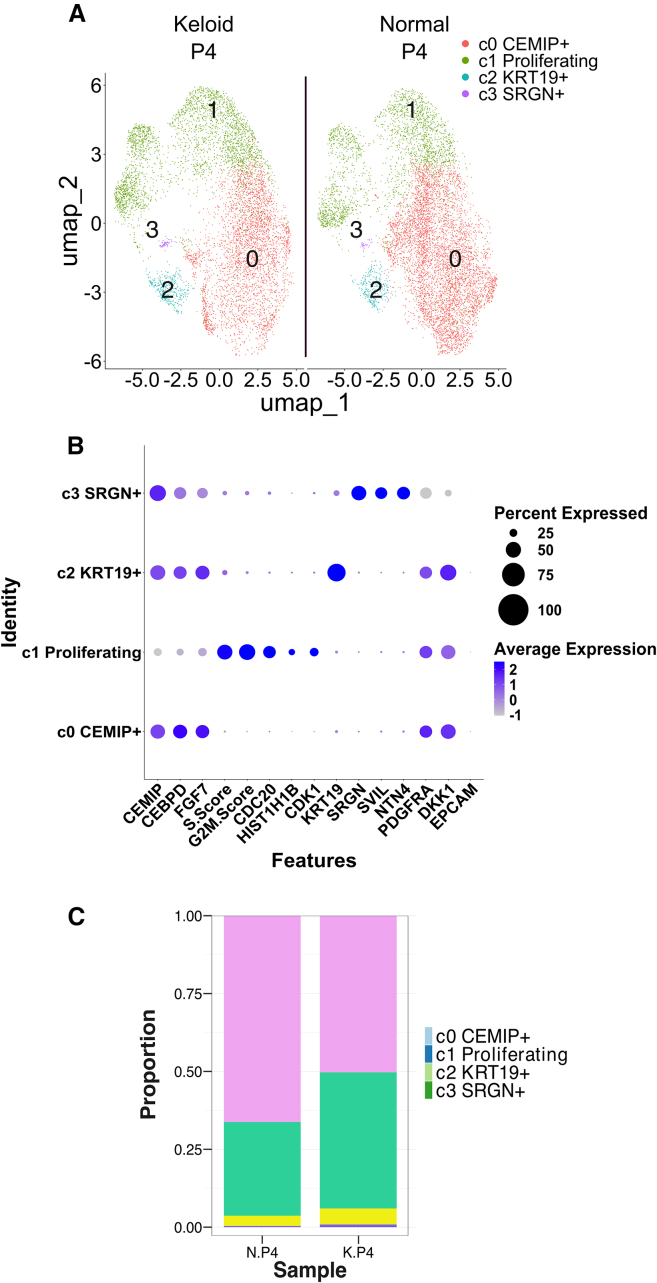
Passage 4-only sub-clustering reveals stromal heterogeneity still present at later passage (A) Sample-separated UMAPs of the unsupervised clustering of 15,255 cells revealed 4 clusters. (B) Expression of cluster marker genes across each cluster confirms cell identity. Blue color gradient represents the average expression of the given gene. Circle size represents the percentage of cells within a cluster expressing a given gene. (C) Proportion of each subset out of total cells for normal (N.P4) and keloid primary dermal cells (K.P4) at passage 4.

**Figure 3:**
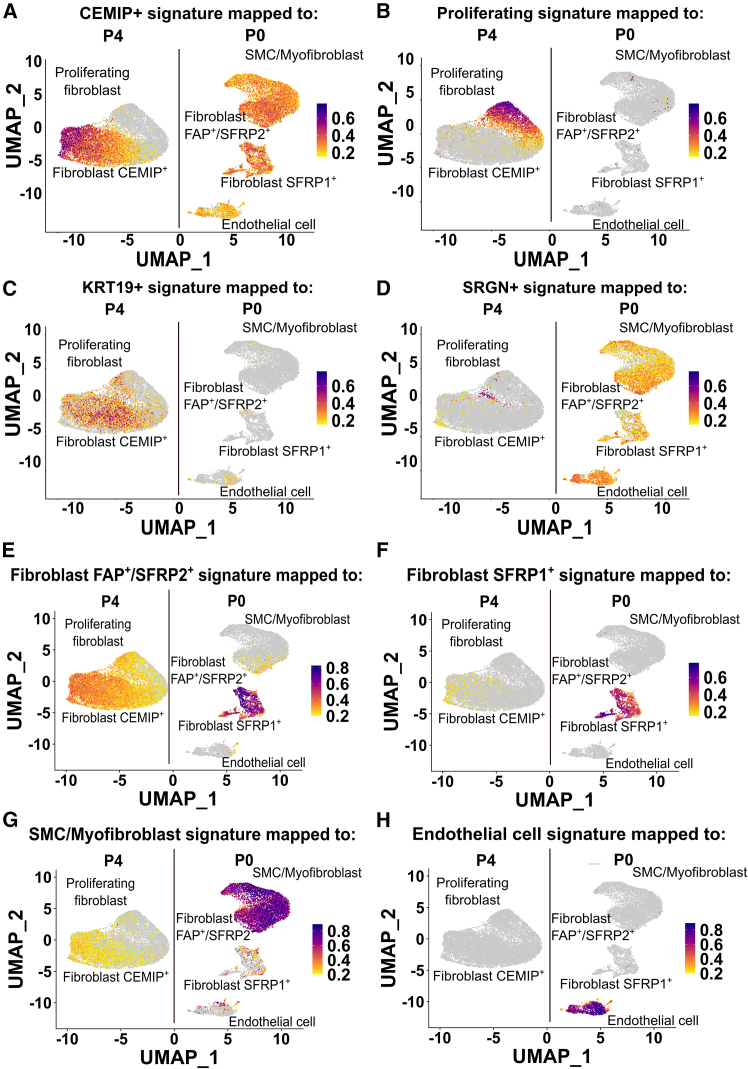
Passage 4 cells represent an amalgamated or common intermediate phenotype of passage 0 stromal cell subsets (A–H) Feature plots show the P4 cluster scores of (A) CEMIP+, (B) proliferating, (C) KRT19+, (D) SRGN+, and P0 cluster scores of (E) Fibroblast FAP+/SFRP2+, (F) Fibroblast SFRP1+, (G) SMC/Myofibroblast and (H) Endothelial cell subsets mapped to the UMAP of the P0/P4 stromal dataset. Grey-purple color gradient represents increasing cluster score (gene signature similarity) of the given subset, per each cell. Plots created using the scCustomize package.

We next increased the granularity of our analysis to investigate the potential for subset heterogeneity after culture; selective re-clustering of P4 cells revealed four subsets (Figure 2A), annotated as CEMIP^+^, proliferating, KRT19^+,^ and SRGN^+^ (Figure 2B). Subset proportions varied slightly between groups, with cultured keloid cells containing more proliferating, KRT19^+,^ and SRGN^+^ cells (Figure 2C). To determine which P0 cell clusters were best represented by the P4 cultures, if any, cluster scores were calculated based on “signatures” (Data S1) using the UCell package (Figure 3). Interestingly, CEMIP^+^ fibroblasts shared a degree of transcriptomic similarity across all P0 stromal subsets, indicating they may represent a common intermediate, or a culture-induced mix (Figures 3A and 3E–3G). Similarly, the P4 SRGN^+^ cells, though representing only a small population, mapped to all P0 clusters, including strongly to endothelial cells, a cell type that classically requires specialized culture supplements to be maintained (Figure 3D). Their hybrid signature (Data S1) potentially corroborates other reports of endothelial cell plasticity in keloid (e.g., EndoMT,^15^ or mesenchymal activation of keloid endothelial cells^16^). Proliferating and KRT19^+^ fibroblasts appeared to be culture-specific phenomena, sharing very little similarity to any P0 subset (Figures 3B and 3C). Together, these data suggest that while some fibroblast states may persist or converge during culture, others, particularly the proliferating and KRT19^+^, likely emerge as culture-induced adaptations.

**Figure 4:**
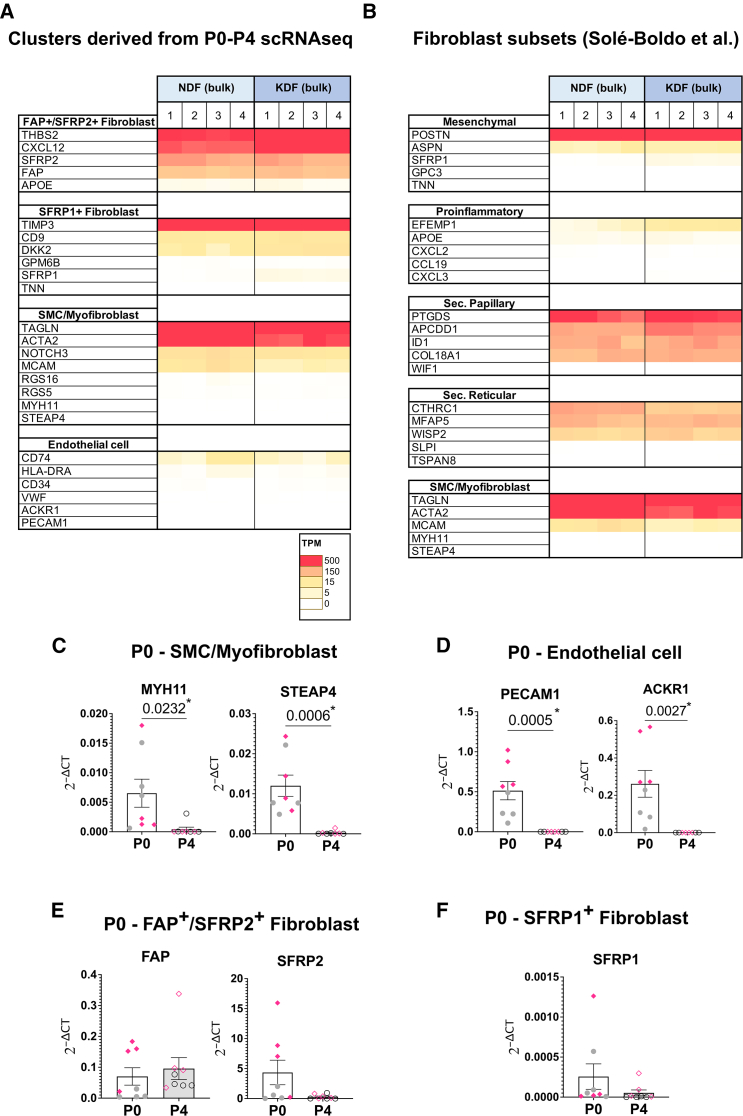
Confirmation of assimilated phenotype in further expanded dermal cell samples (A and B) Raw transcripts per million (TPM) expression of (A) P0 stromal or (B) skin fibroblast subset markers (Solé-Boldo et al.^17^) in patient-matched bulk RNA sequenced keloid versus adjacent non-lesional fibroblasts from P8-9. (C–F) Validation experiments using additional patient samples; *N* = 8/group, with each data-point derived from a different donor. Expression of passage (P) 0 stromal subset markers in P0 (white bar) and P4 (gray bar) normal (gray circles) and keloid (pink diamonds) dermal cells. qPCR expression normalized to the reference gene, GAPDH. Bars represent mean ± SEM. Statistical test: unpaired *t* test; *p*-values are displayed on the graph.

As this single cell RNA sequencing study represented a single biological and technical repeat, the observed changes in cell composition after culture were further investigated by analyzing bulk-RNA-seq for the expression of subset transcriptional marker genes in four additional higher-passage samples (P8-9) from the same donor. In keeping with our scRNA-seq results, P0 cluster markers were largely decreased or lost in high-passage samples, across both keloid and non-lesional groups (Figure 4A). In keeping with our scRNA-seq data, FAP^+^/SFRP2^+^ subset markers were the most maintained, highlighting this subset as the most represented at later passage. Furthermore, to align our data to widely used fibroblast nomenclature, we ascertained subset marker expression first introduced by Solé-Boldo et al.^17^ (Figure 4B). As expected, some markers were decreased or absent in P8/P9 samples, with secretory-papillary and secretory-reticular subsets most represented.

To validate our findings in additional primary patient samples and cultures, we investigated passage changes in cell composition using transcript abundance of marker genes as a surrogate, assessed by qPCR in four unmatched samples of normal skin (from non-keloid individuals) and keloid lesions, again comparing P0 to P4 (Figures 4C–4F). As described above, many P0 subset markers were decreased in P4 samples, and this was observed in both normal and keloid dermal cell cultures. However, there was interesting variability; for example, SFRP2 expression was downregulated, whereas FAP expression was maintained contrary to the P0/P4 scRNA-seq (Figure 4E), indicating that transcriptional changes are not global to the entire subset signature. Similarly, qPCR for some P4 cluster markers validated our sequencing data (e.g., CEMIP, CDK1, and KRTAP1-5 were more highly expressed at P4), while others were not induced (CEBPD, MKI67) or were unexpectedly *less* abundant (SRGN) (Figure S1). Donor variability and the keloid-prone nature of the controls in the initial experiments are two variables potentially contributing to the differences in results between the original single-patient sample pair and the expanded study. Nonetheless, there is notable uniformity in the changes to subset marker expression, indicating a consistency in over-arching fibroblast subtype changes.

### Differential expression in keloid mesenchymal fibroblasts is not fully represented in passaged cells

**Figure 5:**
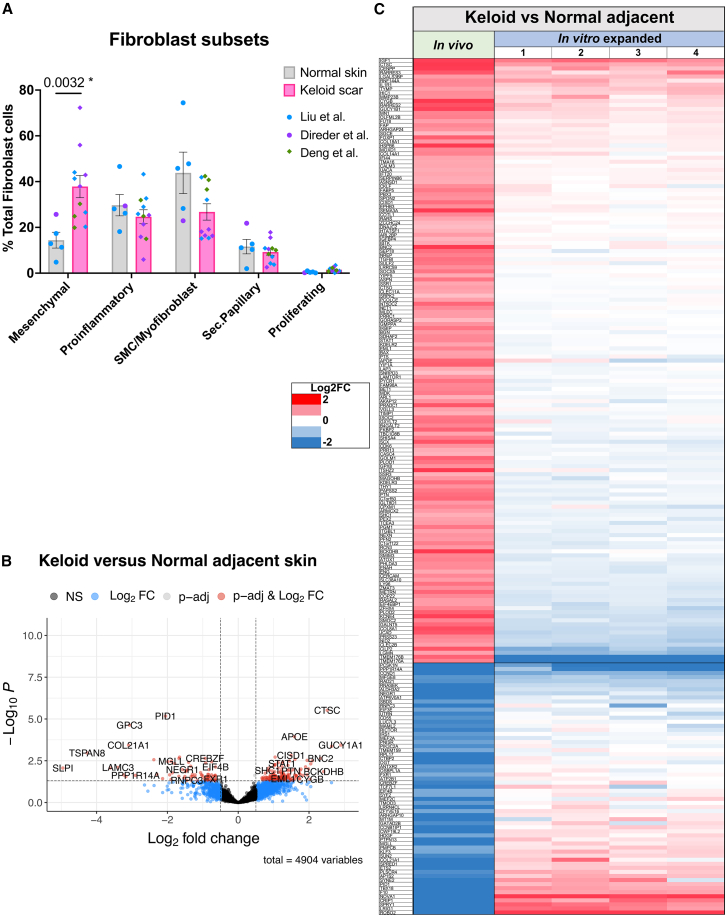
Disease-associated differential gene expression in keloid versus normal mesenchymal fibroblasts is diminished in passaged cells (A) Proportion of fibroblast subsets out of total fibroblast cells for normal skin (gray, *N* = 5 biopsies) and keloid scar (pink, *N* = 11 biopsies; from different individuals). Data derived from Liu et al. (blue points), Deng et al. (green points), and Direder et al. (purple points) scRNA-seq datasets. Statistical test: unpaired, non-parametric *t* test (Mann-Whitney) for which significant *p*-values are displayed on the graph. Bars represent mean ± SEM. (B) Volcano plot displays differentially expressed genes for keloid (*N* = 11 biopsies) versus normal adjacent skin (*N* = 4 biopsies) mesenchymal fibroblasts. Adjusted P cutoff: <0.05, Log2FC cutoff: >0.5 or < -0.5. Plots created using EnhancedVolcano package in R. (C) Heatmap shows Log2 fold change (Log2FC) values of differentially expressed keloid mesenchymal fibroblasts genes in *invivo* scRNA-seq keloid mesenchymal fibroblasts and bulk RNA sequenced keloid fibroblasts from P8-9 (1–2 = P8, 3–4 = P9).

Keloids represent an extreme fibrotic condition, and the fibroblast subsets that they contain differ not only in proportion but also in their transcriptional profiles compared to normal skin. The integration of keloid scar and normal skin samples from three published scRNA-seq datatsets^5^^,^^6^^,^^7^ showed that mesenchymal fibroblasts are significantly increased in keloids compared to other subsets (Figure 5A). Focusing on this subset, we used pseudo-bulk counts and carried out differential gene expression analysis to identify the keloid-specific mesenchymal phenotype (Figure 5B). In total, 227 genes (log_2_FC < −0.5 or >0.5, padj<0.05) were significantly differentially regulated in mesenchymal fibroblasts from keloids (*n* = 11 samples) compared to normal skin (*n* = 4 samples), including known cartilage-related genes such as ASPN and BGN, as previously noted in proteomics analysis,^18^ and other markers (e.g., MDK and SULF2) previously shown to be specific to keloid mesenchymal fibroblasts compared to normal scar^6^ (Data S1). To assess whether these keloid-specific differences persist through passaging, we identified how many of the genes differentially expressed in the pseudo-bulk mesenchymal fibroblast comparison (227 DEGs) can still be observed in the bulk RNA-sequencing of P8/P9 patient-matched keloid versus non-lesional fibroblasts (Figure 5C). 211 of the 227 genes were confidently expressed at P8/9 (TPM count threshold of >1 in all samples in normal skin or keloid group), but only 26 and 8 were differentially up- or down-regulated (adj. *p* < 0.05) in these samples from a single donor. This may suggest the loss of disease-associated features of mesenchymal fibroblasts through culture, acquisition of fibrotic features by non-lesional cells, or inherently fewer differences when comparing keloid cells to adjacent non-lesional fibroblasts than to normal non-keloid-prone controls. Of note, the bulk-RNA sequencing still uncovered significant differential expression between keloid and normal fibroblasts (296 DEGs; log_2_FC < −0.5 or >0.5, padj<0.05), indicating some disease-related differences continue or are highlighted by the culture conditions (Data S1).

### Strategies for the preservation of fibroblast cell subsets across passage

**Figure 6:**
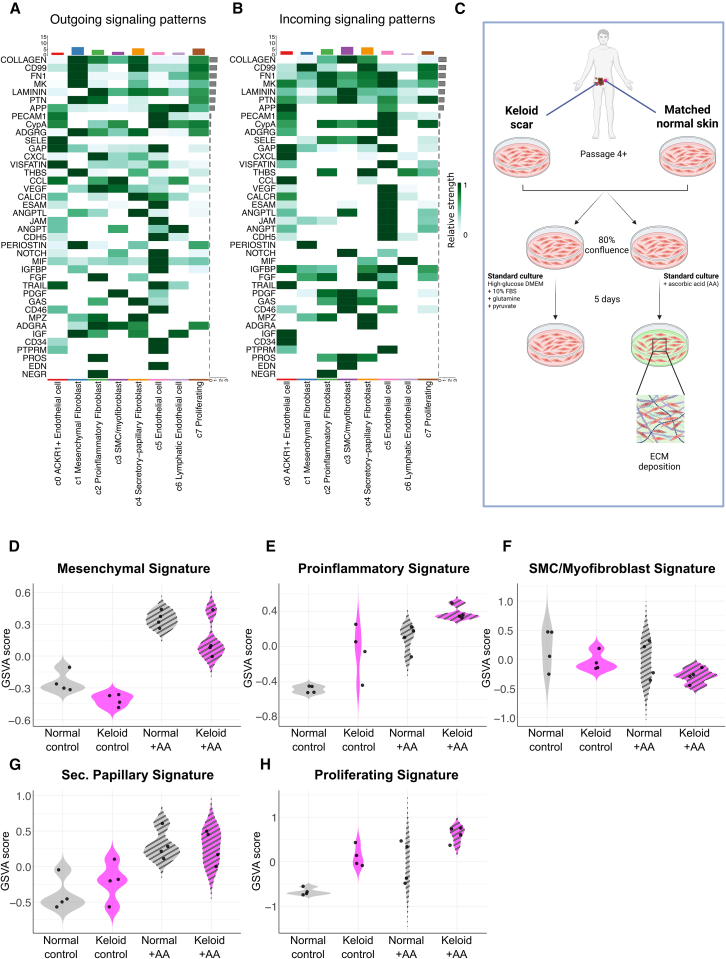
Addition of ascorbic acid to fibroblast culture is sufficient to shift fibroblasts toward a more mesenchymal phenotype (A and B) Heatmap shows the top 25 (A) outgoing and (B) incoming signaling pathways of stromal cell subsets in keloid scars. The green color bar represents the relative signaling strength of pathways across cell subsets, with darker green indicating higher strength (row-scaled values). The colored bar plot at the top shows the total signaling strength of each cell subset, summarizing all pathways. The right gray bar plot indicates the total signaling strength of each pathway, summarizing all cell subsets. Generated using the CellChat package in R. (C) Schematic outlining experimental procedure. Matched keloid and normal skin cells at P8-9 were seeded and grown in standard fibroblast culture conditions with or without ascorbic acid for 5 days. (D–H) Violin plots show gene set variation analysis (GSVA) scores of (D) mesenchymal, (E) proinflammatory, (F) smooth muscle cell (SMC)/myofibroblast, (G) secretory (Sec.) papillary and (H) proliferating fibroblast gene signatures in bulk RNA sequenced normal and keloid fibroblasts with (+AA) or without ascorbic acid (control). GSVA was completed using the GSVA package in R.

Working toward strategies to overcome the limited *in vitro* longevity of fibroblast subsets, we used bioinformatics to pinpoint signaling pathways that may help sustain their phenotype. We reasoned that heavily utilized incoming signaling pathways *in vivo* (i.e., at P0) may be important; therefore, we used the cell-cell communication package CellChat DB^19^ to identify top incoming (and outgoing) signaling pathways (Figures 6A and 6B). As an example, we again focused on mesenchymal fibroblasts, which showed several enriched pathways relating to matrix interaction (collagen, FN1), tissue regeneration (PTN, MDK), and mesenchymal lineage (periostin, CD99). The strength of matrix interaction and the autocrine signaling loop of periostin (POSTN) via the target receptor pair ITGAV and ITGB3 (Figures 6A and 6B), hinted that interaction with the ECM may be responsible for driving the mesenchymal phenotype, and mimicking this trigger in culture may support their maintenance. Therefore, we evaluated whether stimulating the cells to lay down their own ECM during culture would shift their phenotype. We bulk-RNA sequenced P8/P9 keloid and patient-matched non-lesional fibroblasts that were cultured for 5 days using standard fibroblast conditions with or without supplementation with ascorbic acid, a necessary co-factor for collagen synthesis and other ECM component deposition^20^^,^^21^ (Figure 6C). Using the gene signatures of fibroblast subsets from our integrated scRNA-seq analysis (Data S1), we showed the mesenchymal signature was stronger in samples cultured with ascorbic acid (Figures 6D–6H), indicating ECM production and interaction shifts fibroblasts toward a more mesenchymal-like phenotype.

## Discussion

Here we aimed to address key unknowns about primary dermal fibroblast culture, specifically the cell subtypes represented, cell heterogeneity (if any), the transcriptional changes induced by culture, and whether disease-associated molecular alterations are maintained.

As our understanding of fibroblast subtypes based on scRNA-seq across health and disease is still relatively new,^13^^,^^14^ culture-induced changes at this level have only been minimally considered. We used scRNA-seq to examine the changes in cell representation and proportion using a patient-matched pair of non-lesional and keloid dermal cells immediately following tissue dissociation (at P0, referred to as “*in* *vivo*”) and after four passages in standard culture conditions (P4)—a time-point selected to represent established and stable cultures. Although this part of the study relied on primary tissue and cells from a single donor, due to the within-subject design, we were able to isolate changes due to the culturing process alone. As expected, we found non-fibroblast cells were not retained, as we specifically enriched for plastic-adherent cells in conditions typical for fibroblast culture (high-glucose DMEM with 10% serum (single lot used); no specialized growth factors or substrates). Next, focusing on stromal cells only, we observed stark changes; at P0, three distinct fibroblast subtypes were present: FAP^+^/SFRP2^+^, SFRP1^+^, and SMC/myofibroblasts, with FAP^+^/SFRP2^+^ akin to the SPARC^+^/COL3A1^+^ universal fibroblast subtype, while SFRP1^+^ corresponded to the PI16^+^ universal subset.^13^^,^^14^ Both FAP^+^/SFRP2^+^ and SFRP1^+^ subsets also expressed markers of perturbed/pathological fibroblasts^14^: LRRC15/POSTN/COL3A1 and LRRC15/PI16, respectively (Figure S2). Yet by P4, cell phenotypes had dramatically changed and no longer clustered with P0 counterparts. P4-only clustering revealed four distinct clusters: CEMIP^+^, proliferating, KRT19^+^, and SRGN^+^, and this was equal for both non-lesional- and keloid-derived cells. Although CEMIP^+^ and proliferating cells constitute the overwhelming majority (>90%), this diversity shows the cultures are not completely homogeneous, as has been previously suggested^22^^,^^23^^,^^24^; however, their functional differences and the plasticity between clusters remain to be determined. Mapping the P4 cluster signatures to P0 subsets to understand which of the original populations are represented in the culture showed their markers dispersed, suggesting they represent a convergence, or common intermediate, of distinct P0 stromal subpopulations. Such a culture-induced intermediate has previously been reported for joint fibroblasts.^25^ These changes in subset identity are consistent with phenotypic changes across cell culture passage reported by bulk sequencing^12^ and flow cytometry.^26^ Yet, this does not capture the persistent functional differences reported between cultured fibroblast populations such as our work describing fibrosis-related cell behavior of cell and ECM alignment, which is remarkably persistent through passages in culture.^3^ This highlights the importance of understanding the characteristics of models being used and the value in developing strategies to recapitulate specific *in vivo* cell features.

The strength of scRNA-seq is to characterize the presence and proportions of cell subtypes. Here, we confirmed that mesenchymal fibroblasts are abnormally enriched in keloid scars versus normal skin. This is consistent with the original individual reports,^5^^,^^6^ and supports a possible role for these cells in the pathology. Building on decades of studies showing that fibroblasts isolated from keloid and other fibrotic tissues are transcriptionally distinct from controls, we also aimed to explore disease-associated transcriptional differences within specific fibroblast subsets, which can be examined using pseudo-bulk approaches. Here, we focused on the mesenchymal fibroblast population and detected hundreds of differentially expressed genes between keloid and normal skin mesenchymal fibroblasts. Yet, most of these differences were not present in the cultured patient-matched pair of keloid and non-lesional cells. This finding indicates that comparing cells using standard *in vitro* conditions may miss and/or underestimate biological differences within certain cell subsets. However, we still detected 295 differential genes in cultured keloid cells versus non-lesional controls, indicating many disease-relevant characteristics persist or emerge. For example, SOCS3, a known STAT-response gene and downstream target of IL-6 signaling, was upregulated in cultured keloid cells, which further supports our previous data that autocrine IL-6 signaling is uniquely active in cultured keloid fibroblasts.^3^ Additional studies powered to detect within-cell-type, disease-associated transcriptional differences are needed. Moreover, future work could address what features of the culture system (e.g., serum,^27^ substrate stiffness,^28^^,^^29^ and absence/presence of specific supplements) are particularly influential on subtype survival and on inducing differential expression in established cultures, and indeed, whether they equally affect normal, non-lesional, and keloid fibroblasts. Here we only compared pre-culture (P0) to passage 4+, time-points postulated to represent a stable typical fibroblast “monoculture.” It would be a valuable addition to also analyze: 1) intermediate time-points (e.g., P1-P3) to determine the duration of survival for non-fibroblasts (and their influence), and the speed at which the phenotypic changes triggered by the culture conditions are observed; and 2) incremental later time-points to understand the stability of the *in vitro* characteristics. We hypothesize that the attrition of non-stromal cell populations would be variable and influenced by the starting tissue composition and handling, but that the fibroblast-specific culture-induced changes would be, in part, rapid (akin to the <24h serum response reported by Iyer et al.^27^), with some features taking longer to stabilize (e.g., the mechano-response of primary human dermal fibroblasts is described by Achterberg at 7 days^30^). Given these time frames, we speculate that primary fibroblast cultures from P4+ (and potentially earlier) can offer consistent and valuable models, albeit with strengths and limitations.

With an aim to develop an *in vitro* model to investigate the mesenchymal cellular phenotype in keloids, strategies to maintain this fibroblast subset were explored. Others have reported that positional signals can retain fibroblast subtype identity; for example, Wei et al.^25^ showed that synovial sublining versus lining fibroblasts are maintained by NOTCH signaling from nearby endothelial cells. We used cell-cell communication analysis to identify heavily utilized incoming signaling pathways. Periostin was identified as an autocrine signal for mesenchymal fibroblasts, consistent with previous reports (Figure S3).^6^ We particularly noted periostin as an important incoming signal for P4 CEMIP^+^ fibroblasts, yet the main source would be the SRGN^+^ population, which at this passage is much-reduced (Figure S4). Along with other ECM interaction pathways (collagen, fibronectin), this indicated that keloid-relevant mesenchymal fibroblasts might be retained, or their phenotype encouraged, by the presence of ECM in the culture conditions. We tested this using ascorbic acid, an important cofactor needed for collagen secretion and the stabilization of other ECM components.^20^^,^^21^ As hypothesized, adding ascorbic acid into standard culture conditions for 5 days resulted in a transcriptional shift toward a mesenchymal phenotype. Similarly, transitions toward more *in vivo*-like phenotypes with cultures inclusive of skin ECM derivatives have been described,^31^ but this has not included in-depth transcriptional analysis of individual subset markers. Furthermore, the powerful transcriptional effects of the ECM have been demonstrated previously with mesenchymal stem cells, with differing compositions leading to distinct cell lineages.^32^^,^^33^^,^^34^^,^^35^ Though the cell-derived ECM in this study was not characterized, future studies could evaluate whether keloid-specific ECM components^18^^,^^36^ are stably expressed in established cultures and if they may have sustaining effects on keloid cell phenotypes.

There are now many commercially available media formulations specific for primary fibroblasts (e.g., FGM-2 and FibroGro; some supplemented with FGF, insulin) that may alter these findings again; our data may serve as a resource, highlighting marker genes for characterizing cells resulting from different expansion conditions. Here, we use ascorbic acid and, in turn, the ECM as an example of a wide-ranging incoming signal that could potentially enrich or maintain mesenchymal fibroblasts. This is just one example, but it is hoped that the findings can also be exploited for other fibroblast subsets; e.g., neuronal growth regulator (NEGR) manipulation may uniquely promote a proinflammatory phenotype (Figure 6B).

In conclusion, this study uses transcriptional information to describe the specific fibroblast subset changes that occur after multiple passages in culture. This increased understanding of our model system is required for investigating fibroblast cell biology, from the functional differences between subpopulations to the pathological mechanisms in the context of keloid scarring and fibrosis. Furthermore, we describe a potential method for adjusting culture conditions to promote a specific phenotype. As the field works toward increasingly sophisticated *in vitro* models of disease (e.g., 3D, complex organoids), knowledge of how culture components may affect cell identity is important. Future work dedicated to establishing protocols to isolate and preserve distinct subsets will be valuable, as accurate models are required for scientific understanding and the development of much-needed novel therapeutics.

### Limitations of the study

This study characterized the culture-induced changes to dermal fibroblasts grown for 4 passages under standard conditions. This is a single time-point that we propose represents a practical, established phase of culture. However, it does not capture the temporal dynamics of any changes nor their stability.

Single-cell sequencing was performed on non-lesional (keloid-prone) and lesional (keloid) tissue from a single donor, which limits our ability to report on disease-associated differences, such as differentially expressed genes in specific cell populations or the cellular composition of tissues at the time of isolation. However, the experimental design did allow us to discover a collective culture-induced shift in fibroblast phenotype, which was validated in more donor samples via qPCR.

We propose theoretical manipulations to the culture system that might influence/maintain fibroblast subpopulations or their features; however, it was beyond the scope of this study to test these experimentally.

## Resource availability

### Lead contact

Further information and requests for resources and reagents should be directed to and will be fulfilled by the lead contact, Tanya Shaw (tanya.shaw@kcl.ac.uk).

### Materials availability

This study did not generate new unique reagents.

### Data and code availability

• Data: Generated scRNA-seq and bulk RNA-seq data are available in NCBI’s Gene Expression Omnibus (GEO) and accessible through accession numbers GSE303591 (bulk RNA-seq) and GSE293834 (scRNA-seq). Publicly available scRNA-seq data are accessible by the following: Liu et al.^5^: HRA000425, hosted on the Genome Sequence Archive (GSA); Deng et al.^6^: GSE163973, hosted in the GEO repository; Direder et al.^7^: GSE181316, hosted in the GEO repository. Accession Codes also available in the key resources table.
• Code: Code to reproduce the analyses described in this manuscript can be found in the Data S2 in (∗.html) and (∗.rmd) formats.
• Additional information: Any additional information required to reanalyze the data reported in this paper is available from the lead contact upon request.

## Acknowledgments

AL was supported by the 10.13039/501100000265UK Medical Research Council (MR/N013700/1) and King’s College London as a member of the MRC Doctoral Training Partnership in Biomedical Sciences. This work was further supported by the MRC IAA 2021 King’s College London (MR/X502923/1; FD,TS,AL) and the 10.13039/100010269Wellcome Trust (107859/Z/15/Z; BS).

ED was supported by King’s College London as part of the Cell Therapies & Regenerative Medicine Wellcome Trust PhD Program (108874/Z/15/Z).

The graphical abstract was created in BioRender. Lock, A. (2026)https://BioRender.com/qhth5mp.

For the purpose of Open Access, the author has applied a CC BY public copyright license to any Author Accepted Manuscript (AAM) version arising from this submission.

## Author contributions

Conceptualization: A.L., B.S., T.S., and F.D.; data curation: A.L.; formal analysis: A.L.; funding acquisition: T.S. and F.D.; investigation: A.L.; methodology: A.L., D.F., T.S., and F.D.; project administration: T.S. and F.D.; resources: A.L., T.S., F.D., and D.F.; software: A.L.; supervision: T.S. and F.D.; validation: A.L., T.S., and F.D.; visualization: A.L.; writing – original draft preparation: A.L.; writing - review and editing: A.L., T.S., F.D., E.D., D.F., and B.S.

## Declaration of interests

The authors declare no competing interests.

## Declaration of generative AI and AI-assisted technologies in the writing process

Nothing to declare.

## STAR★Methods

### Key resources table

**Table:** 

REAGENT or RESOURCE	SOURCE	IDENTIFIER
**Biological samples**		

Primary human tissue/cells – details in Table S1	N/A	N/A

**Chemicals, peptides, and recombinant proteins**		

Iodinated povidone (0.025g/10mL)	Ecolab	3030440
Gentamycin solution (0.05mg/mL)	Sigma Aldrich	G1397
Dispase II	Gibco	17105041
Human Whole Skin Dissociation Kit	Miltenyi Biotec,	130-101-540
HyClone^TM^ Fetal Bovine Serum (FBS)	Cytiva	SV30160.03, Lot RF20200007
DMSO	Sigma Aldrich	D2650
High-glucose Dulbecco’s Modified Eagle Medium (DMEM with GlutaMAX and pyruvate)	Gibco	31966021
Penicillin/streptomycin solution (100x)	Sigma-Aldrich	P0781
Trypsin-EDTA solution (10X, 0.5% w/v)	Sigma-Aldrich	T4174
MACS Dead Cell Removal Kit	Miltenyi Biotec	130-090-101
RLT buffer	Qiagen	74104
Beta-mercaptoethanol solution	PanReac AppliChem	A1108.0100
Gelatin (from bovine skin)	Sigma-Aldrich	G9391
Glutaraldehyde solution	Alfa Aesar	A10500.22
Glycine	Sigma-Aldrich	G8790
Ascorbic acid	Sigma-Aldrich	A4403
RNeasy Mini kit (for bulk RNA sequencing)	Qiagen	74104
RNeasy Micro Plus kit (for RT-qPCR)	Qiagen	74034
SuperScript III reverse transcriptase (200U/μL)	Thermo Fisher Scientific	18080093
Random primers	Invitrogen	48190011
dNTPs (for RT-qPCR)	Invitrogen	18427013
SYBR Green I Master Mix	Roche	04887352001
*For Smart-seq2*		
SuperScript II Reverse Transcriptase	Thermo Fisher Scientific	18064014
UltraPure DNase/RNase-Free Distilled Water	Thermo Fisher Scientific	10977035
dNTP Mix; 10mM each	Thermo Fisher Scientific	10319879
TE buffer, pH 8.1, RNAse free	Thermo Fisher Scientific	15445059
MgCl_2_ (1 M) AM9530G	Thermo Fisher Scientific	AM9530G
TRITON(R) X-100 BIOXTRA	Sigma-Aldrich	T9284-100ML
BETAINE, 5M	Sigma-Aldrich	B0300-1VL
DNA-OFF	*Takara Bio*	9036
Recombinant RNase Inhibitor	*Takara Bio*	2313A
KAPA HiFi HS RM (100 x 25 μl rxns)	Roche	07958927001

**Critical commercial assays**		

Chromium Single Cell 3′ Reagent Kit v3.1	10X Genomics	PN-1000128
Qubit high sensitivity RNA assay	Invitrogen	Q32852
Bioanalyzer 2100 (for bulk RNA sequencing)	Agilent	5067-1529
Tapestation 4150 (for RT-qPCR)	Agilent	5067-5576-78

**Deposited data**		

Bulk RNA-seq data	GEO repository	GSE303591
scRNA-seq	GEO repository	GSE293834

**Oligonucleotides**		

Primers for RT-qPCR, see Table S3	This paper	N/A
*For Smart-seq2*	N/A	N/A
ISPCR oligo: AAG CAG TGG TAT CAA CGC AGA GT, 200 nmole, HPLC purified	Thermo Fisher Scientific	N/A
oligo-dT(30)VN: AAG CAG TGG TAT CAA CGC AGA GTA CTT TTT TTT TTT TTT TTT TTT TTT TTT TTT TTA G, 50 nmole, PAGE purified	Thermo Fisher Scientific	N/A
TSO Custom LNA Oligonucleotide, RNA oligo (250 nmole) purified by RNase-Free HPLC Purification: AAGCAGTGGTATCAACGCAGAGTACATrGrG+G	Exiquon now Qiagen	339412

**Software and algorithms**		

kb-python package	Andre et al.^37^	N/A
Seurat R package version 4.3.0	Butler et al.^38^ Satija et al.^39^ Stuart et al.^40^	N/A
UCell R package	Andreatta et al.^41^	N/A
CellChat package (v 1.5.0)	Jin et al.^19^	N/A
DESeq2 in R	Love et al.^42^	N/A
EnhancedVolcano package in R	Blighe et al.^43^	N/A
GSVA R package	Hänzelmann et al.^44^	N/A
GraphPad Prism version 10.2.0	Graphpad.com	N/A
SOAPnuke	github.com/BGI-flexlab/SOAPnuke	N/A

**Other (publicly available datasets used)**		

Liu et al.^5^ scRNA-seq data	Genome Sequence Archive (GSA)	HRA000425
Deng et al.^6^	GEO repository	GSE163973
Direder et al.^7^	GEO repository	GSE181316

### Experimental model and study participant details

#### Human subjects

Human sample collection was approved by the North of Scotland Research Ethics Committee (REC)(reference: 14/NS/1073). Informed written consent was obtained from all participants. See Table S1 for donor demographics (sex, age, sample location, allocation to groups). The central “core” of the keloid lesions (distant from scar margin) was used for all keloid cell isolations. For patient-matched studies, the adjacent margin tissue was used for “non-lesional” cell comparisons. Normal (non-keloid prone) samples were processed from full-thickness skin as provided following plastic surgery procedures.

#### Cell culture

##### Tissue dissociation

Tissue samples were washed successively in 10% w/v iodinated povidone (0.025g/10mL, Ecolab, #3030440), 70% ethanol (Sigma Aldrich, #32221) and twice in 0.05mg/mL gentamycin solution (Sigma Aldrich, #G1397). Sub-cutaneous fat was then removed with a scalpel, but otherwise the full depth of the tissue was used. Tissue was first soaked in 10mg/mL Dispase II (Gibco, #17105041; solution prepared in Hank’s Balanced Salt Solution) overnight at 4°C. Then, epidermis was removed by peeling/scraping, and the remaining dermis was dissociated using a Human Whole Skin Dissociation kit (Miltenyi Biotec, #130-101-540) as per manufacturer’s instructions. The resulting cell suspension was frozen at -80°C in complete media with 40% fetal bovine serum (FBS; Cytiva HyClone^TM^, #SV30160.03) and 10% DMSO (Sigma Aldrich, #D2650) before being moved to vapour-phase nitrogen until culture.

### Method details

#### Experimental fibroblast culture

Normal (adjacent non-lesional and non-keloid prone) and keloid dermal cells were defrosted and grown until 80% confluency in 2D on Nunc® cell culture plastic surfaces (supplied by Thermo Fisher Scientific) in ‘complete medium’: high-glucose Dulbecco’s Modified Eagle Medium (DMEM with GlutaMAX and pyruvate, Gibco, #31966021) + 10% FBS (Cytiva, #SV30160.03, consistent single lot: RF20200007) and penicillin/streptomycin (Sigma-Aldrich, #P0781; 100 units penicillin and 0.1mg streptomycin per mL final concentration). Cells were passaged according to standard protocols, detaching them by incubation with trypsin-EDTA solution (0.5% w/v, diluted to 0.05% in PBS; Sigma-Aldrich, #T4174) for 5 mins at 37°C.

For P0/P4 scRNA-seq, P0 vials of normal and keloid dermal cells were thawed and then cultured in complete medium over four passages. These cells (P4) were then frozen at -80°C in complete medium with 40% FBS and 10% DMSO before being moved to vapour-phase nitrogen. Immediately prior to sequencing, both P0 and P4 vials were thawed and enriched for live cells using the MACS Dead Cell Removal Kit (Miltenyi Biotec, #130-090-101).

To study the effects of matrix deposition on fibroblast phenotype, a protocol based on cell-derived matrices was adapted and utilised from Kaukonen et al.^45^ First, sterile 13 mm diameter glass coverslips (VWR, #631-1578) were added to a 24 well plate (VWR, #734-1605) and rinsed in 70% ethanol for at least 15 minutes. After PBS washing, sterile 0.2% (wt/vol) gelatin (from bovine skin, Sigma Aldrich, #G9391) was added for 60 minutes at 37°C. Coverslips were then rinsed and the gelatin crosslinked with 1% glutaraldehyde (Alfa Aesar, #A10500.22) for 30 minutes at room temperature. Coverslips were rinsed with sterile PBS and incubated with 1M Glycine (Sigma Aldrich, #G8790) for 20 minutes at room temperature to quench crosslinking. Coverslips were rinsed with PBS and incubated with complete medium for 30 minutes at 37°C and either used immediately, or were filled with at least 1mL of PBS and stored for up to 1 month at 4°C.

Normal and keloid dermal fibroblasts (P8-P9) were thawed and seeded in triplicate at a density of 26,300 cells/cm^2^ (24-well plate, near confluency) and left overnight to adhere and spread. Cells were cultured in complete medium with or without supplementation with freshly prepared 50μg/mL ascorbic acid (Sigma Aldrich, #A4403) for 5 days, with media changes every other day. This experiment was repeated on serial passages, P8 – P9, which were used as technical replicates (N=4).

For bulk RNA sequencing and qPCR experiments, cultures were lysed directly in the plates in 350μL RLT buffer (Qiagen, #74104), supplemented with 1% beta-mercaptoethanol (PanReac AppliChem, #A1108.0100) before being frozen at -80°C until downstream analysis.

#### RNA extraction and quantification

RNA was purified using either the RNeasy Mini kit (for bulk RNA sequencing) (Qiagen, #74104) or the RNeasy Micro Plus kit (for RT-qPCR) (Qiagen, #74034) following manufacturer’s instructions and quantified using a Qubit high sensitivity RNA assay (Invitrogen, #Q32852). RNA quality was ascertained by either Bioanalyzer 2100 (Agilent) (for bulk RNA sequencing) or Tapestation 4150 (Agilent) (for RT-qPCR). In both cases, RNA Integrity Number (RIN) values above 9 were used.

#### Single cell RNA sequencing - Library preparation and sequencing

RNA from P0/P4 matched samples was given to the Waterloo Genomics Centre, King’s College London for generation of single-cell gel beads, barcoding, clean up, cDNA amplification and library construction with a Chromium Single Cell 3′ Reagent Kit v3.1 (10X Genomics, #PN-1000128). The resulting libraries were sequenced on an Illumina HiSeq 2000 platform (Illumina) by Novogene (Novogene Co. Ltd, Cambridge, UK).

#### Single cell RNA sequencing - Data analysis

Raw sequencing fastq files were processed using the kb-python package (Melsted et al.^37^). Briefly, a pseudoalignment index was created using the *Homo sapiens* Ensembl reference transcriptome (GRCh38.p14). Read matrices were converted to Seurat objects using the Seurat package (v4.3.0)(Butler et al.,^38^ Satija et al.,^39^ Stuart et al.^40^), allowing for quality control (QC) and analysis (authors’ standard workflow); QC filters applied: nFeature_RNA <5000 & >500, percent.mt <15. All subsequent analyses were performed using Seurat R package version 4.3.0, with detailed scripts provided in ’Supplementary_Script’. Processed and raw data are available under GEO accession GSE293834, with the following Seurat objects: all P0/P4 data before (GSE293834_processed.Robj.gz) and after SCTransform (GSE293834_sct_v4.Robj.gz); the stromal cell subsets only (GSE293834_sct_v4_stromal.Robj.gz) as well as the P4 only subset (GSE293834_P4_only.Robj.gz). See also Data S1 for normalization parameters. Clusters emerging from the unsupervised analysis were assigned cell types by cross-referencing the top 15 DEGs (by proportion) with the Panglao^46^ and Human Protein Atlas^47^ online databases. The P0/P4 dataset did not require integration, as samples were run on the same 10X chip in batch-controlled fashion. Previously published keloid scRNA-seq datasets^5^^,^^6^^,^^7^ were computationally integrated in Seurat by identifying variable features across individual biopsies (Figure S5). Stromal subset signature scores (P0 and P4) were calculated using the UCell package.^41^ Selection of signature genes is described in Data S1. Finally, the CellChat package (v 1.5.0)^19^ was used to analyse the integrated dataset, following the authors’ vignette.

To identify a keloid mesenchymal fibroblast transcriptional phenotype, pseudobulk counts were generated from the integrated scRNA-seq stromal-only Seurat object, which was further subsetted to include only mesenchymal fibroblasts from keloid and normal skin samples (excluding normal scar). Pseudobulk counts were generated by first normalising counts using the ‘RC’ method (Seurat V4) and then summing across all cells per sample. Differential gene expression analysis was performed in R using DESeq2,^42^ and visualised with volcano plots generated using the EnhancedVolcano package.^43^ The resulting gene list was then filtered for expressed genes (basemean>50) and for significant (padj<0.05) upregulated (Log2FC>0.5) and downregulated (Log2FC<-0.5) genes.

#### Bulk RNA sequencing - Sample preparation

RNA from P8-9 normal and keloid fibroblasts cultured with and without ascorbic acid for 5 days were shipped to BGI Genomics Co. Ltd., Hong Kong for library preparation and sequencing using the BGISEQ platform. The SOAPnuke software was used to filter out low-quality reads, with remaining clean reads aligned to the reference human genome (Homo Sapiens GRCh37) and using HISAT2 and Bowtie2 software. All fastq and processed files are available on the GEO repository under accession number GSE303591. Transcripts per million (TPM) values were used for analyses in Figures 4A, 4B, and 6D–6H. For comparison to pseudobulk differential expression in Figure 5C, raw read counts from our bulk-RNA seq were normalised using the same method as DESeq2 (estimateSizeFactors) and then filtered for truly expressed genes (TPM>1 in all samples in normal skin or keloid group).

#### Bulk RNA sequencing – Data analysis

*Comparison to ex vivo keloid mesenchymal fibroblast differential expression:* To evaluate whether P8-9 keloid fibroblasts maintained *ex vivo* keloid mesenchymal differential gene expression, Log_2_FC values from the bulk sequenced keloid control (i.e. absence of ascorbic acid) samples were compared to the pseudobulk-DESeq2 outputs from keloid versus normal skin mesenchymal fibroblasts. Log_2_FC values for each bulk RNA sequenced keloid control sample was calculated based on comparison to the mean average normalised read count value of the normal control group.

*Gene set variation analysis (GSVA):* To evaluate the change in cell phenotype with and without ascorbic acid treatment, gene set variation analysis (GSVA) was performed using gene signatures of *ex vivo* fibroblast subsets derived from the integrated scRNA sequencing data.^5^^,^^6^^,^^7^ Signatures for each cluster were compiled based on genes that were expressed at Log_2_FC>1.5 or ratio>1.5 (padj<0.05) compared to all other stromal subsets (Data S1). Log TPM values were used for analysis to avoid skewing by extreme values. Analysis was carried out using the GSVA R package.^44^

#### RT-qPCR

RNA was reverse transcribed using SuperScript III reverse transcriptase (200U/μL) with dithiothreitol (0.1M) and first-strand buffer (5X) (Invitrogen, #18080093) and random primers (Invitrogen, #48190011) plus dNTPs (Invitrogen, #18427013) according to manufacturer’s instructions. Due to low RNA yield (<4ng/μL) from some samples, RNA from all samples for the P0-P4 sequencing validation experiment were amplified and converted to cDNA using the Smart-seq2 protocol (steps 1 to 27).^48^ cDNA was diluted to 2ng/μL, and qPCR was performed using a Roche LightCycler 480II with SYBR Green I Master Mix (2x; Roche, #04887352001) according to manufacturer’s instructions. Primer sequences were from KiCqStart (sequences in silico ‘validated’) if available, or designed with primerBLAST (sequences provided in Table S3). Provided by Sigma-Aldrich, all primer pairs were tested for efficiency (80-120 accepted) and specificity (gel electrophoresis).

### Quantification and statistical analysis

#### Statistical analysis

Statistical analysis was performed using GraphPad Prism software v10 or R. Statistical parameters for each analysis can be found in the relevant figure legends.

## Supplemental information

Document S1. Figures S1–S5 and Tables S1–S3

Data S1. Excel File containing bioinformatic analysis outputs, related to all Figures

Data S2. ZIP file containing analysis code in (∗.html) and (∗.rmd) formats
