# Supplementary material for: Single-cell RNA-sequencing of dermal fibroblasts demonstrates culture-induced changes and variable persistence of keloid disease features
Source: iScience. 2026 Apr 7;29(5):115630. doi: 10.1016/j.isci.2026.115630 (PMC13123497; doi:10.1016/j.isci.2026.115630)
Supplement: Document S1. Figures S1–S5 and Tables S1–S3 [file mmc1.pdf]

iScience, Volume *29*

Supplemental information

Single-cell RNA-sequencing of dermal fibroblasts demonstrates culture-induced changes and variable persistence of keloid disease features

Amy Lock, Elena M. Drudi, Dasha Freydina, Brian M. Stramer, Franziska Denk, and Tanya J. Shaw

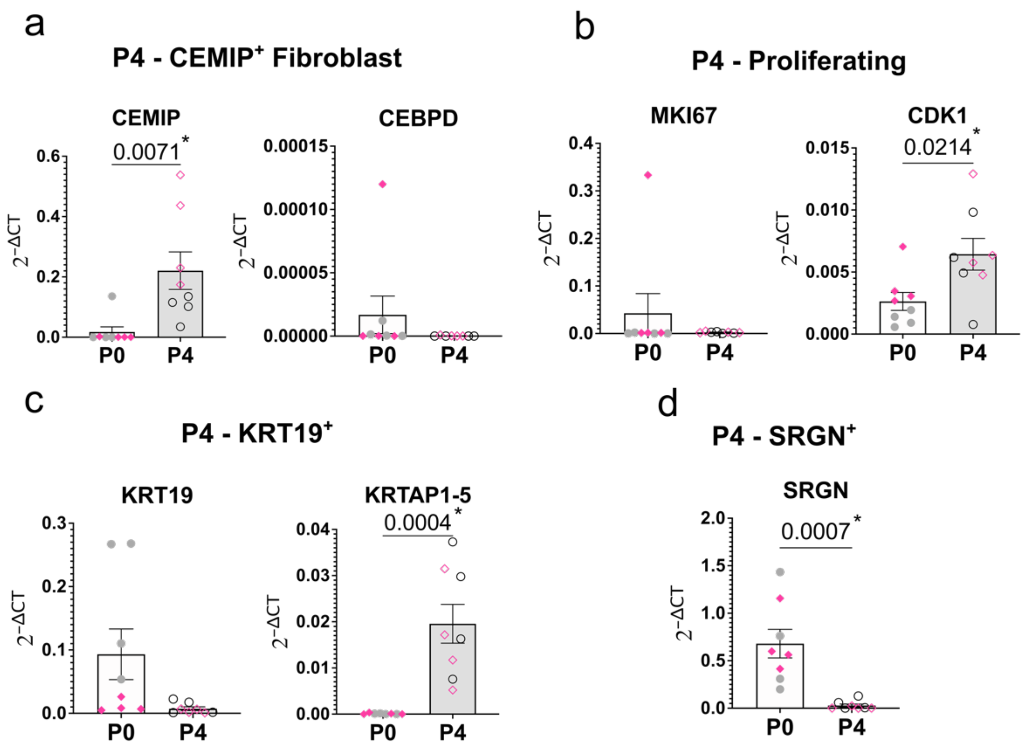

Figure S1. Confirmation of P4 cell subsets in further expanded dermal cell samples.

(a-d) Expression of passage (P) 4 subset markers in P0 (white bar) and P4 (grey bar) normal (grey circles) and keloid (pink diamonds) dermal cells. N=8/group, with each dot derived from a different donor. qPCR expression normalized to reference gene, GAPDH. Bars represent mean +/- SEM. Statistical test: Unpaired T-test; p-values are displayed on the graph.

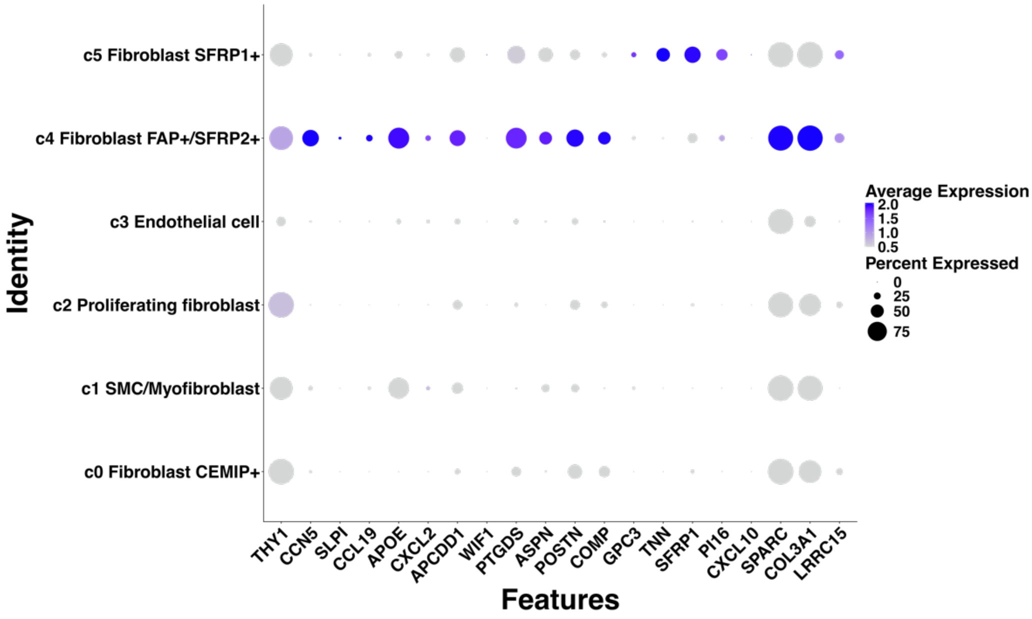

Figure S2. Expression of subset specific and universal fibroblast markers in P0/P4 stromal cells.

Dot plot showing the expression of marker genes across each stromal cluster in the P0/P4 dataset. Blue color gradient represents average expression of given gene. Circle size represents the percentage of cells within a cluster expressing a given gene.

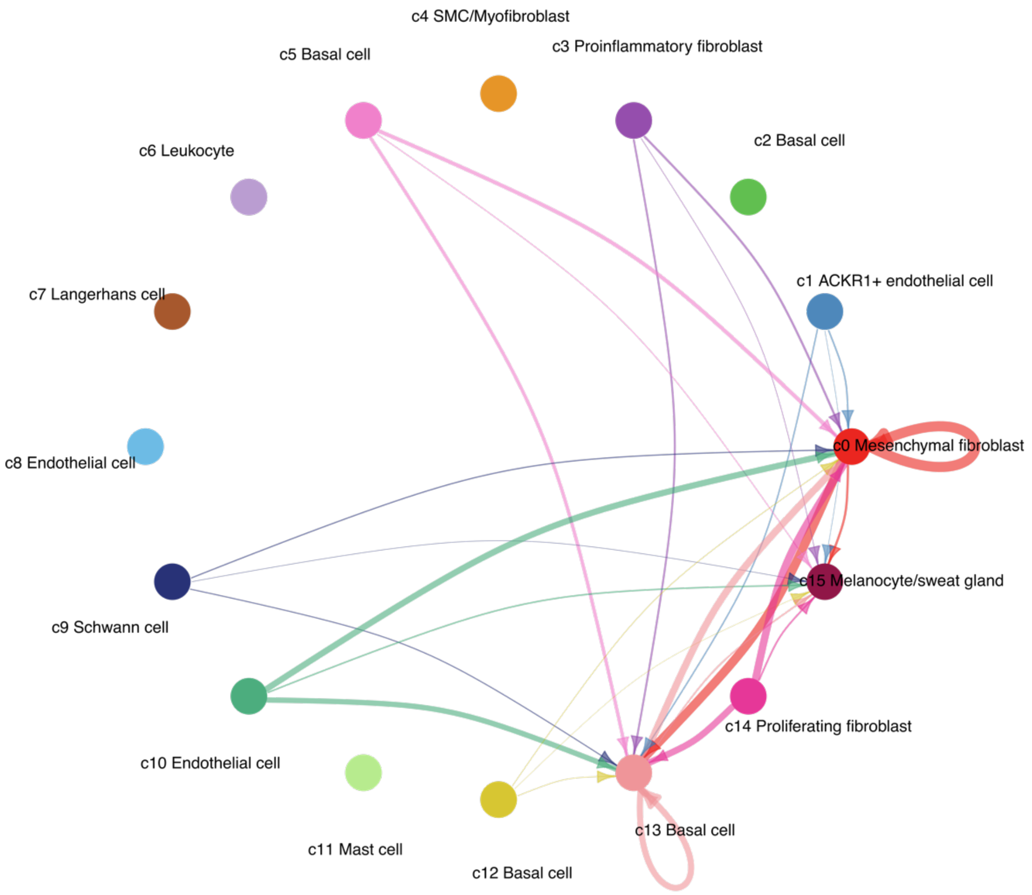

Figure S3. Mesenchymal fibroblasts may receive periostin signaling from several cell types and may act in an autocrine signaling loop.

Analysis using data derived from integrating previously published scRNA-seq studies of keloid and normal skin/scar (see Methods). Circle plot illustrating the periostin signaling network (POSTN-ITGAV:ITGB3). Arrowheads point toward receivers (direction of signaling input). The thickness of lines represents the strength of signal. Plot created using the package CellChat.

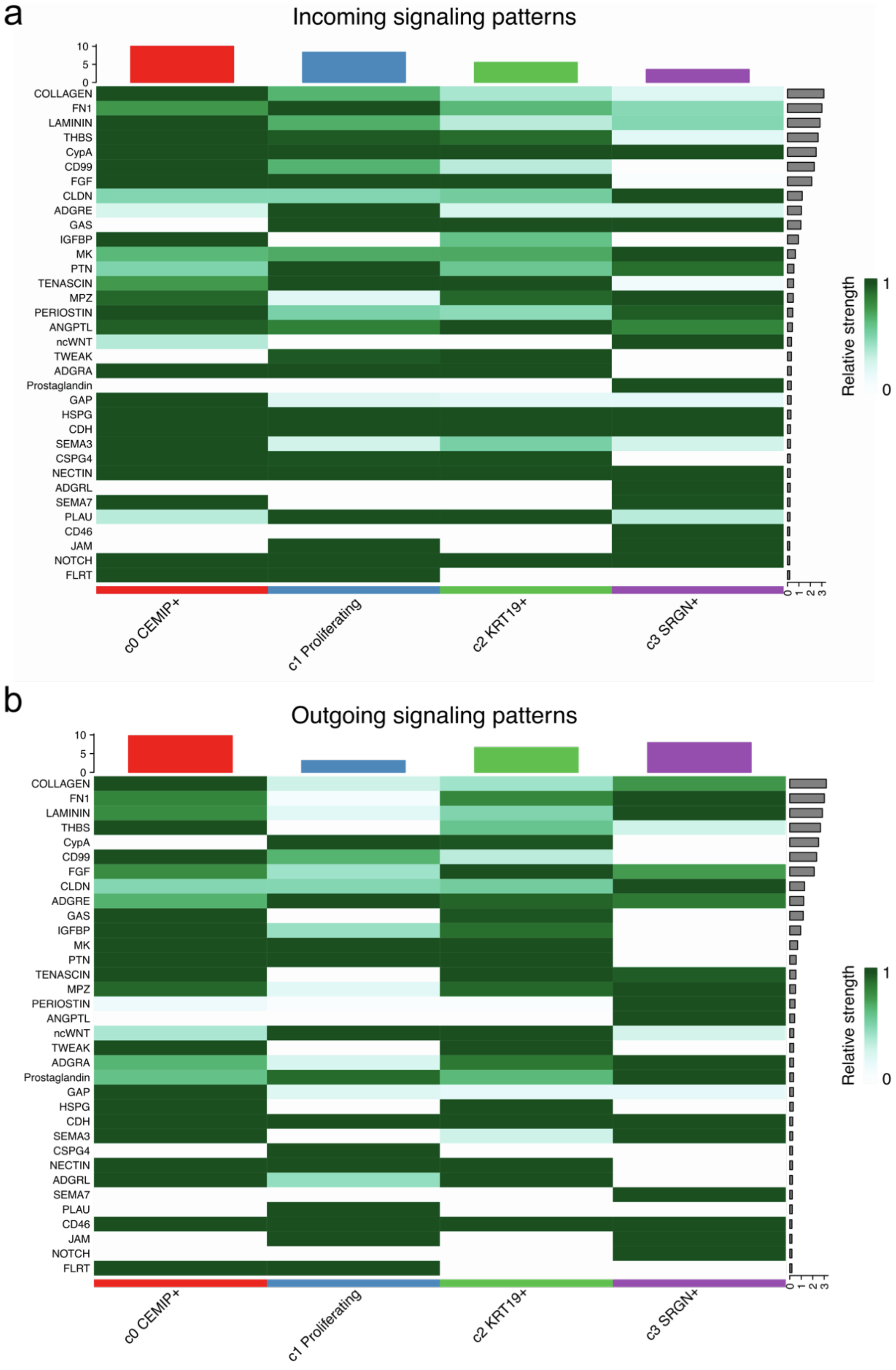

Figure S4. Cell-cell communication analysis highlights potential pathways preserved at later passage culture.

(a-b) Cell-cell communication analysis of (a) incoming and (b) outgoing signals within P4-only cell clusters. The green color bar represents the relative signaling strength of pathways across cell subsets, with darker green indicating higher strength (row-scaled values). The colored bar plot at the top shows the total signaling strength of each cell subset, summarizing all pathways. The right grey bar plot indicates the total signaling strength of each pathway, summarizing all cell subsets. Generated using the CellChat package.

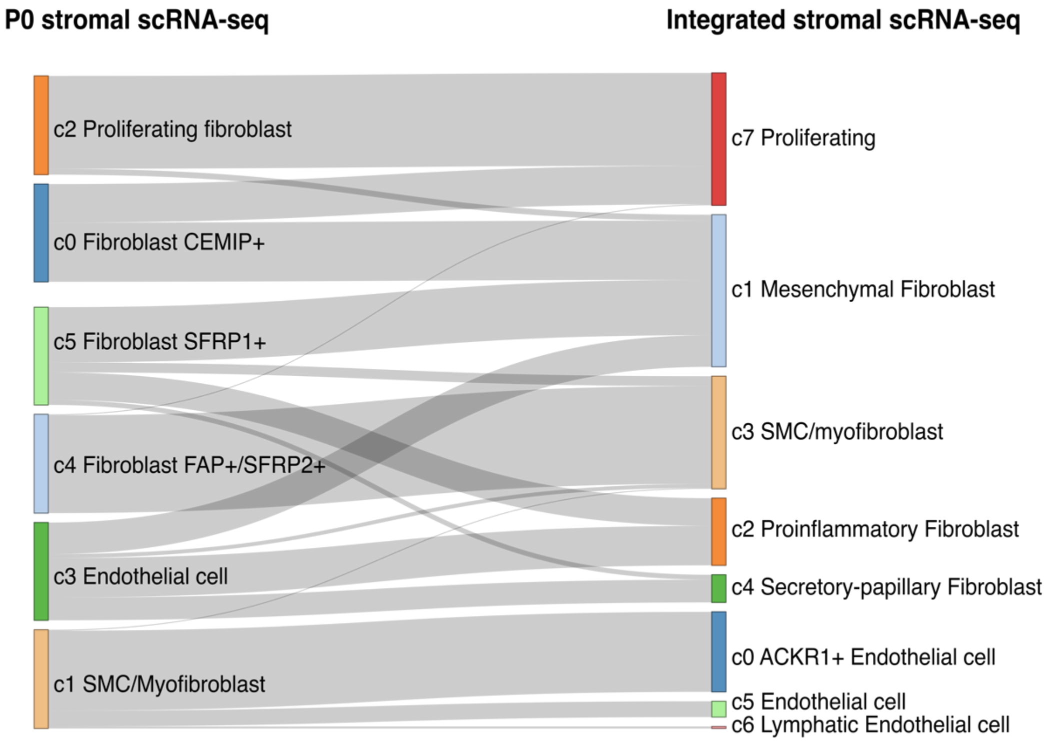

Figure S5. Mapping of P0 annotated stromal subsets to integrated scRNA-seq dataset. Sankey plot illustrates how P0/P4 stromal subsets identified in this study map to the integrated stromal dataset clusters. The thickness of lines is proportional to the number of cells.

Table S1. Donor demographics

Internal Sample ID	Normal/Keloid	Gender	Age at excision	Scar location	Passage
P0/P4 scRNA-seq and P8/P9 bulk RNAseq (Figure 1-3, Figure 4a-b, Figure 5c, Figure 6d-h)					
240216	Normal & Keloid (paired)	M	53	Pelvis	0 & 4<br>8 & 9
P0/P4 Normal skin/Keloid scar samples (Figure 4c-f - qPCR validation)					
280616B	Keloid	F	UNKNOWN	Ear	0
170215	Keloid	F	24	Ear	0 & 4
180216	Keloid	F	50	Abdomen	0
090316	Keloid	F	50	Umbilicus	0
011015A	Keloid	M	38	Back	4
240915	Keloid	UNKNOWN	UNKNOWN	UNKNOWN	4
181111	Keloid	UNKNOWN	UNKNOWN	UNKNOWN	4
090915A	Normal	F	19	Breast	0
251115	Normal	UNKNOWN	UNKNOWN	UNKNOWN	0 & 4
160517	Normal	UNKNOWN	UNKNOWN	Thigh	0 & 4
300915	Normal	F	44	Back	0 & 4
020915	Normal	F	22	Breast	4

Table S2. scRNA-seq analysis parameters

Analysis	Prinicipal components used for RunUMAP	Resolution used for FindClusters	Normalisation
Liu et al dataset [1]– Overall clusters	15	0.5	Log
Deng et al [2] – Overall clusters	15	0.4	Log
Direder et al [3] – Overall clusters	20	0.3	Log
Integrated dataset – Overall clusters	20	0.2	Log
Integrated dataset – Stromal clusters	15	0.2	Log
P0-P4 dataset - Overall clusters	20	0.2	SCTransform
P0-P4 dataset - Stromal clusters	20	0.3	SCTransform
P0-P4 dataset – P4 clusters	20	0.15	SCTransform

1. Liu, X., Chen, W., Zeng, Q., Ma, B., Li, Z., Meng, T., Chen, J., Yu, N., Zhou, Z., and Long, X. (2021). Single-cell RNA-seq reveals lineage-specific regulatory changes of fibroblasts and vascular endothelial cells in keloids. J Invest Dermatol. 10.1016/j.jid.2021.06.010.
2. Deng, C.-C., Hu, Y.-F., Zhu, D.-H., Cheng, Q., Gu, J.-J., Feng, Q.-L., Zhang, L.-X., Xu, Y.-P., Wang, D., Rong, Z., and Yang, B. (2021). Single-cell RNA-seq reveals fibroblast heterogeneity and increased mesenchymal fibroblasts in human fibrotic skin diseases. Nature Communications *12*, 3709. 10.1038/s41467-021-24110-y.
3. Direder, M., Weiss, T., Copic, D., Vorstandlechner, V., Laggner, M., Pfisterer, K., Mildner, C.S., Klas, K., Bormann, D., Haslik, W., et al. (2022). Schwann cells contribute to keloid formation. Matrix Biol *108*, 55-76. 10.1016/j.matbio.2022.03.001.

Table S3. RT-qPCR Primers

Gene	Forward	Reverse
GAPDH	GGAAGGTGAAGGTCGGAGTCAAC	CAGAGTTAAAAGCAGCCCTGGT
ACKR1/DARC	AAGGATGGTCTTCTCATCTG	TTTTCACAAAGGCAGTGTAG
CDK1	ATGAGGTAGTAACACTCTGG	CCTATACTCCAAATGTCAACTG
CEMIP	ACCGAGCACATTCCAACTACCG	GGCAGAGATGATTGAGAGGAACG
CEBPD	CAGACTTTTCAGACAAACCC	TTTCGATTTCAAATGCTGC
FAP	GAAGAGGAAATGCTTGCTAC	CTAGGATATTGTTCATCGCC
KRT19	AACCATGAGGAGGAAATCAG	CATGACCTCATATTGGCTTC
KRTAP1-5	AGTTCTCAGACTTTGCATTG	TTTGTAGCATTTCTGTGTCC
MKI67	GACAGAGGTTCCTAAGAGAG	AACAATCAGATTTGCTTCCG
MYH11	CTATCTGCTAGAAAAATCACGG	CACTTCTCATCTTCTCCTTG
SFRP1	CTTAAGTGTGACAAGTTCCC	TTTTCATCCTCAGTGCAAAC
SFRP2	GACCTAGACGAGACCATC	ATACCTTTGGAGCTTCCTC
SRGN	GAATCCTCAGTTCAAGGTTATC	GATCTTGTTGGATTCACCTG
STEAP4	TGATTCATATGTGGCTTTGG	CAGTTTGGACTGGACAAATC
PECAM1	TGGAAAGCAGATACTCTAGAACGG	GGGATGTGCATCTGGCCTT
